# 

**Published:** 1889-06

**Authors:** 


					﻿JTJKTE 1S£9.
OLD SERIES
Vol. xxv
NEW
Vol. UA
& 1855 o
WiTkANTA

JttmE



W. F. WESTMORELAND, M D
H. V. M, MILLER, M. D. LL, D.
VIRGIL 0, HARDON, M. D.
Published By james p.harrison&cq.
i» _______ Atlanta,ga. £&
SUBSCRIPTIONS S2.QO A YEAR
				

